# Autonomous Corrosion Assessment of Reinforced Concrete Structures: Feasibility Study

**DOI:** 10.3390/s20236825

**Published:** 2020-11-29

**Authors:** Woubishet Zewdu Taffese, Ethiopia Nigussie

**Affiliations:** 1 Department of Civil Engineering, University of Aalto, 02150 Espoo, Finland; woubishet.taffese@aalto.fi; 2 Department of Future Technologies, University of Turku, 20014 Turku, Finland

**Keywords:** sensors, internet of things, intelligent data analytics, machine learning, deep learning, autonomous corrosion assessment, corrosion, reinforced concrete

## Abstract

In this work, technological feasibility of autonomous corrosion assessment of reinforced concrete structures is studied. Corrosion of reinforcement bars (rebar), induced by carbonation or chloride penetration, is one of the leading causes for deterioration of concrete structures throughout the globe. Continuous nondestructive in-service monitoring of carbonation through pH and chloride ion (Cl^−^) concentration in concrete is indispensable for early detection of corrosion and making appropriate decisions, which ultimately make the lifecycle management of RC structures optimal from resources and safety perspectives. Critical state-of-the-art review of pH and Cl^−^ sensors revealed that the majority of the sensors have high sensitivity, reliability, and stability in concrete environment, though the experiments were carried out for relatively short periods. Among the reviewed works, only three attempted to monitor Cl^−^ wirelessly, albeit over a very short range. As part of the feasibility study, this work recommends the use of internet of things (IoT) and machine learning for autonomous corrosion condition assessment of RC structures.

## 1. Introduction

Reinforced concrete (RC) structures represent a large proportion of the world’s physical infrastructure with great economic importance. Building and infrastructure accounts for 47% of the world gross domestic product [1]. Despite their contribution to the economy, a high number of infrastructure in industrialized countries are approaching the end of their service life and suffer from considerable degree of deterioration [2,3]. For example, the US physical infrastructure is graded as D^+^ (one scale above from the least grade) by American Society of Civil Engineers in 2017, indicating that the infrastructure is generally at risk [4]. 

Corrosion of reinforcement bars (rebar) is one of the major causes for degeneration of RC structures throughout the world, especially those exposed to aggressive environments [5,6,7]. Studies have demonstrated that corrosion of rebar has already reached an alarming level and the associated maintenance and repair costs exceed multibillion USD per year globally. In the developed countries corrosion cost is estimated in the range of 3.5–4.5% of their GDP [8]. Continued rebar corrosion in repaired RC structures is even responsible for 37% of the failure modes [9], causing costly repairs [9,10,11].

Naturally, concrete cover provides a high level of protection to the embedded rebar against corrosion since the pore solution of the concrete is alkaline with a pH of 12–13. Under this amount of alkalinity, rebar persists passivated. However, the passivation of rebar is breakdown due to the presence of Cl^−^ or by the carbonation of the concrete [12,13,14]. Carbonation is a natural physicochemical process triggered by the penetration of CO_2_ from the neighboring environment into the concrete through pores in the matrix where the CO_2_ reacts with hydrated cement [15,16]. Although the reduction of alkalinity caused by carbonation changes the chemical composition of concrete, its major out-turn is that it destroys the passive oxide layer of rebar, which eventually initiates corrosion [13,15,17,18,19]. The penetration of Cl^−^ is also the primary cause for rebar corrosion in concrete environment. Like carbonation, the penetration of Cl^−^ does not typically cause harm directly to the concrete. Nonetheless, when the amount of Cl^−^ concentration at the rebar reaches a certain threshold, depassivation occurs that cause corrosion initiation [20,21,22,23,24]. Indeed, the chloride threshold levels are considerably scattered over a wide range of values (from close to zero to about 2.5 wt% Cl^−^ by mass of binder) as it is controlled by several factors including composition of the concrete, the type of rebar, and the exposure conditions [25,26]. Both, carbonation- and chloride-induced corrosion, dwindle the cross-sectional area and the resistance of the rebar. This causes severe cracking as well as reduction in the load-bearing capacity of the infrastructure. Cracked concrete allows an additional entry of moisture, aggressive gasses, and ions, aggravating rebar corrosion and concrete degradation. Subsequently, the serviceability, strength, and safety of the infrastructure are impacted adversely.

Corrosion initiation time must be precisely estimated to carry out in-time and cost-effective maintenance decisions to prevent RC structures from corrosion-induced damage. To do so, continuous assessment of pH of the concrete and the amount of Cl^−^ concentrations in the concrete is crucial. Measurement of pH of the concrete can indicate the risk of corrosion caused by carbonation. The conventional methods for evaluating carbonation and the Cl^−^ content in concrete is often semi-destructive, time-consuming, and demand periodic repetition [27]. In these methods, the depth of carbonation and the amount of Cl^−^ concentrations in the concrete are determined using chemical and/or physical laboratory tests of samples taken from in-service structures. Due to these facts, the conventional techniques cannot be applied for frequent measurements. In addition, performance investigation of RC structure either in the field or laboratory (by taking sample from the structure) usually incur a high amount of direct and indirect costs [28]. For instance, traffic delay costs caused by conventional field inspection and maintenance programmes are valued to be in the range of 15–40% of the construction costs [29]. After evaluating the carbonation front and the amount of chloride concentration from the samples, prediction of the remaining lifetime of the structure is usually carried out using conventional empirical models. These models are based on Fick’s second law of diffusion and comprises several assumptions and simplifications, inducing considerable uncertainty in predicting the time to onset rebar corrosion [30]. This uncertainty could have serious effect on inspection and maintenance planning, which in turn adversely influence the service life of the structure and escalate the life-cycle costs. Therefore, reliable, cost-effective, and in-service monitoring of pH and Cl^−^ concentration in concrete along with accurate corrosion assessment method is indispensable, which ultimately makes the service-life management of RC structures efficient from economy and safety perspectives. 

The objective of this work is to carry out feasibility study on autonomous assessment of rebar corrosion due to carbonation and Cl^−^ penetration in RC structures. The contributions of this work are threefold: (i) in-depth review of state-of-the-art Cl^−^ and pH sensing devices that can be embedded in concrete structures, (ii) study on the potential of IoT system for continuous Cl^−^ and pH monitoring, and (iii) a critical insight into the integration of IoT systems and intelligent data analytics to achieve autonomous corrosion assessment of RC structures. 

The structure of the remainder of the paper is as follows. In Section 2, various types of sensing devices that are proposed for monitoring Cl^−^ and pH in concrete environment are critically reviewed. The possibility of integrating the physical infrastructure with digital technologies by employing internet of things based continuous Cl^−^ and pH monitoring system is presented in Section 3. In Section 4, the future smart infrastructure that autonomously assess the corrosion condition of RC structures is proposed. Finally, conclusions are provided in Section 5.

## 2. State-of-the-Art Chloride and pH Sensors

The risk of structural disintegration of infrastructure caused by rebar corrosion fosters the development of sensing devices for continuous nondestructive monitoring that enable early detection of corrosion. Over the past years, embeddable sensors have been developed and installed in concrete structures for monitoring the corrosion state of rebar. There are even commercially available corrosion monitoring systems based on different approach including measurements of macrocell, resistivity, and electrochemical polarization dynamics. The drawback of these types of sensors is that they provide information about the corrosion state of the rebar after corrosion has initiated. While sensors that can monitor pH of the pore solution and Cl^−^ concentration can deliver information about the state of the rebar embedded in the concrete well before corrosion initiates, serving as early alerting systems. Hence, this paper focuses in reviewing only Cl^−^ and pH sensors that monitor chloride concentration and pH of the concrete pore solution. In addition, pH and Cl^−^ sensors can be used for controlling concrete repair works. For example, repair work to passivate rebar using electrochemical chloride extraction and re-alkalization methods [31].

To perform a thorough study on the recent development of Cl^−^ and pH sensing devices that can be embedded in concrete structures, databases of the Scopus and Web of Science were selected for analysis. Both are abstract and citation databases of peer-reviewed literature, delivering a complete citation search by giving access to numerous databases. A set of queries that comprise the manuscript’s title, topic, abstract, and keywords were carried out on every database in order to choose works reporting Cl^−^ and pH sensing devices that can be embedded in concrete structure. Since one of the focuses is to evaluate the current development Cl^−^ and pH sensors, the lifespan of the search criteria for both databases were set to 10 years. Any duplicated records from the databases were removed and then manually checked to select suitable ones. Table 1 presents the applied search terms, selection criteria and number of records screened and included. A total of 34 scientific papers have been critically reviewed and presented the findings. As can be seen in Table 1, more number of chloride sensors have been proposed compared to pH sensors.

### 2.1. Potentiometric Sensors

In the past few decades, several electrochemical sensors based on potentiometric ion-selective electrodes (ISEs) have been developed to monitor change of Cl^−^ concentrations in concrete. ISEs are firmly established and have been adopted in several areas. For instance, in the area of analytical chemistry, ionic activity of some specific species had been examined using a potential measurement [32]. The working principle of potentiometric sensors is based on potential change caused by the chemical activity of the sensed ion with regard to a reference ISE under the conditions of no current flow. Typically, such types of electrodes are primarily membrane-based devices, comprising ion-conducting permeable materials, which isolate the sample from the inside of the electrode [33]. One of the electrodes is the working or indicator electrode whose potential is determined by its environment. The second electrode is a counter or reference electrode which has a fixed potential. Since the potential of the counter electrode is constant, the potential difference value can be associated with the dissolved ion concentration [33]. 

The potential of a potentiometric sensor is estimated using the Nernst equation, described by Equation (1) [24], which determines the linear dependence of the sensor response (E) on the logarithm activity change of the ion in the solution:

(1) $$E=E^{o}+\frac{R\;T}{n\;F}\ln\left[a_{cl^{-}}\right]-E_{ref}$$

where E is the potential difference for the electrochemical cell composed of the ion-selective and reference electrode [V], $E^{o}$ is the standard electrode potential, *R* is the gas constant [${J\;\mathrm{mol}}^{-1}K^{-1}$], T is the absolute temperature [K], F is the Faraday constant [${C\;\mathrm{mol}}^{-1}$], n is the number of electrons involved in the reaction, and $a_{cl^{-}}$ is the chemical activity of the Cl^−^ [${\mathrm{mol}\;\mathrm{dm}}^{-3}$], and $E_{ref}$ is the standard reference electrode potential [V].

The widely used reference electrodes and their potentials with respect to the regular hydrogen electrode (NHE) is given in Table 2. It can be noticed that the unpolarizable reference electrodes (calomel (Hg_2_Cl_2_), silver/silver chloride (Ag/AgCl), manganese dioxide (MnO_2_), copper/copper sulphate (Cu/CuSO_4_)) have constant potential values. All the polarizable electrodes, except lead (Pb), showed potential values with a fluctuation range of ±20 [34,35]. Among all, Ag/AgCl are the most widely used electrodes for measurement of different environments, including concrete [36,37]. This is due to the fact that these electrodes have the following superior characteristics: (i) exhibit potential regulated by a reversible balance, (ii) return to the reversible potential after small polarizations, and (iii) stable and robust [37]. 

#### 2.1.1. Potentiometric Chloride Ion Sensors

Though the fundamental principles of potentiometric ISEs based sensors are well established and widely applied in the field of electrochemistry, their applicability in concrete must be assessed. Sensors that can be mounted in concrete should operate in high alkaline environment and in a wide range of Cl^−^ concentrations for a long time while providing accurate and reliable results. Moreover, the stability of the Cl^−^ sensors to pH changes and other major interfering ions (which come from the environment and/or the concrete ingredients) should be assured. There are studies that have evaluated the performance of potentiometric ISEs in monitoring Cl^−^ concentration in concrete [32,38,39,40,41,42,43,44,45,46,47,48]. 

Ag/AgCl electrodes are the most commonly utilized and commercially available chloride ion selective electrodes [27,43]. It comprises a wire made of silver (Ag) coated with a conversion layer of silver chloride (AgCl). The first attempt to measure activities of Cl^−^ using Ag/AgCl electrodes in hardened mortar was documented in the early 1990s [5]. Since then, several Cl^−^ sensors based on Ag/AgCl electrodes have been developed. Table 3 summarizes the development progress of state-of-the-art Cl^−^ and pH potentiometric sensors and their performance in concrete environment. The performance of the sensors was evaluated using simulated concrete pore solutions, mortar, and concrete.

Femenias et al. [44] investigated the sensitivity of the Ag/AgCl electrodes to other interfering species that can be emanating from the surrounding environment and/or from the concrete mix ingredients. The findings of the investigation demonstrated that insignificant interference from fluoride, sulphate, and hydroxyl but substantial from bromide and sulphide. In completely chloride-free alkaline solutions, the ISEs were not stable over time, but upon arrival of Cl^−^, it reliably measures the Cl^−^ concentration. The authors concluded that Ag/AgCl electrode can satisfactorily be applied to monitor the amount of Cl^−^ in RC structures exposed to chloride-laden environments. Montemor et al. [38] evaluated the suitability of Ag/AgCl electrodes for in-service monitoring of Cl^−^ in concrete environment. The evaluation was performed in mortar and concrete specimens at different depths by exposing to a broad range of Cl^−^ concentrations. The reported results revealed that the Ag/AgCl based chloride sensor is reasonably stable. 

Pargar et al. [41] characterized the response of Ag/AgCl in simulated pore solutions by testing with variable amount of Cl^−^ and pH values. The claimed test results showed that the influence of pH on the potential value of the sensor is trivial at Cl^−^ concentrations of > 4 mM. This makes Ag/AgCl based sensors are practical to monitor the change of Cl^−^ in concrete structures since Cl^−^ concentration of 4 mM is much under than the usually stated thresholds that initiate rebar corrosion in concrete. As the pH has a notable effect on the reading of the sensor at low Cl^−^ concentration (<4 mM), it is vital to monitor the pH value and Cl^−^ concentration simultaneously for accurate measurement of Cl^−^ content. Similar phenomenon was also observed regarding the influence of pH on measuring Cl^−^ concentration in concrete made by another study [32]. Jin et al. [43] applied Ag/AgCl based sensors to monitor Cl^−^ concentration in concrete specimens composed of different mineral admixtures at different depths. On the basis of the potential analysis of the embedded Cl^−^ sensor, the authors concluded that continuous monitoring of Cl^−^ concentration in RC structure could be achieved. They also remarked that extra efforts are required to produce inexpensive, robust, and reliable Cl^−^ potentiometric sensors that can be utilized in concrete environment.

Karthick et al. [45] produce a solid-state alkaline stable polymer-coated Ag/AgCl sensor for monitoring of Cl^−^ concentration in concrete structure. The alkaline stability of the sensor and the coating material were evaluated in synthetic concrete pore solution and characterized using advanced tests, including half-cell potential, scanning electron microscopy (SEM), X-ray diffraction (XRD). The sensor was calibrated in wide range of Cl^−^ concentrations using three different solutions: (i) saturated KCl solution, (ii) distilled water solution, and (iii) synthetic concrete pore solution. Then the sensor was embedded in the concrete at the depths of 5, 10, 20, 30, and 40 mm for 90 days.

Based on the findings, the authors claimed that the sensor has excellent chloride sensing ability and well stable in an alkaline medium, and thus it is suitable to monitor Cl^−^ concentration in concrete structure. Gao et al. [46] produce all-solid-state Cl^−^ sensor employing MnO_2_ and Ag/AgCl electrodes. The potentiometric response and stability of the sensor was examined in synthetic concrete pore solutions with diverse NaCl concentrations ranging from 0.05–5.0 M. The performance of the sensor with the existence of interfering ions of K^+^, Ca^2^^+^, Na^+^, and SO_4_^2^^−^ was tested. The authors reported that the potentiometric response of the sensor to chloride is slightly influenced by the interfering ions, but considerably affected by the pH at low-chloride concentration.

Meanwhile, over the range of 5–45 °C, the sensor’s potential reading linearly grows with the solution temperature. The sensor reveals outstanding polarization behaviour as proven by galvanostatic and potentiodynamic tests. Based on these facts, the author concluded that the sensor has excellent potential for Cl^−^ monitoring in concrete environments. Indeed, the authors recommend the pH of the concrete pore solution to be simultaneously monitored to accurately determine the Cl^−^ in slightly chloride contaminated concrete. Gao et al. [47], in another work, concluded the same after tested Ag/AgCl based potentiometric Cl^−^ sensor in synthetic concrete pore solutions with a wide range of NaCl concentrations ranging from 0.005–1.0 M.

**Figure 1:**
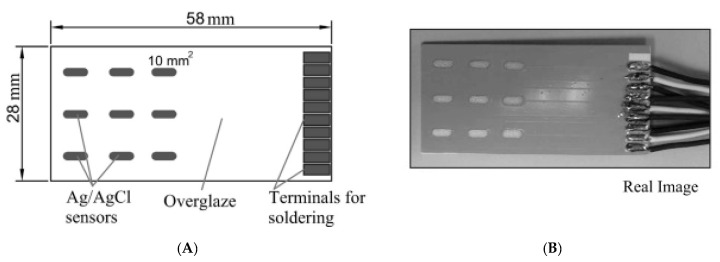
Potentiometric thick-film Cl^−^ sensor arrays: (**A**) substrate dimensions and (**B**) real image [42].

All the above short- and long-term evaluations of potentiometric Ag/AgCl based chloride sensors performed in pore solutions, mortar and concrete specimens demonstrated that Ag/AgCl electrode is stable and reliable in alkaline environments, and thus they could be suitable to monitor Cl^−^ concentrations in concrete structures. However, the main hurdles in realizing these sensors for monitoring concrete structures are the lack of appropriate sensors, which desirably have to be inexpensive, robust, and easily maintainable. Commercially available potentiometric chloride sensors typically utilize ISEs which show durability problems and can be costly. To address these problems, Gandía-Romero et al. [42] developed a potentiometric thick-film (screen-printed) Cl^−^ sensors made of Ag/AgCl resistive pastes, aiming to produce accurate, miniaturized, long-term, and inexpensive electrodes for monitoring Cl^−^ concentration in concrete. This technology is based on a printing method, which comprises a rubber palette to transfer inks via a display on the substrate surface. Screen-printed sensors are generally robust, inexpensive, and allow integration of multiple sensors in a small space. Sensors based on this technology are ideal for industrial mass production since it is possible to utilize resistive pastes, which are readily available in the market. The developed thick-film Ag/AgCl sensors are illustrated in Figure 1. These sensors were tested in concrete specimens. The reported test result demonstrated that the developed thick-film Ag/AgCl sensors could provide reliable and real-time measurements regarding the change of the Cl^−^ concentration in concrete specimens at different depths. The author concluded that the thick-film Ag/AgCl is a promising Cl^−^ sensor for concrete structure since it is robust, miniaturized, low cost, and have long-term stability.

**Figure 2:**
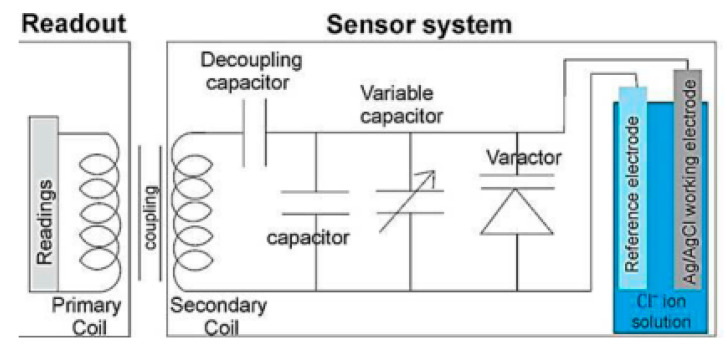
Block diagram of the wireless method for Cl^−^ monitoring using Ag/AgCl electrode [48].

The data transmission method of all the above mentioned potentiometric Cl^−^ sensors is through cables. There are few studies which demonstrate the possibilities of wireless monitoring of Cl^−^ in concrete element using potentiometric sensors. Abbas et al. [48] were able to wirelessly measure the Cl^−^ concentrations using sensor based on Ag/AgCl electrodes. The block diagram of the proposed wireless method for monitoring Cl^−^ using Ag/AgCl electrode is shown in Figure 2. The wireless communication was attained using a near-field-inductive coupling between the coils of the readout and the sensor. The potential of Ag/AgCl electrode is converted into capacitance by considering it as a bias potential for a varactor. The resonance frequency of the sensor coil alters because of the alteration in the capacitance of a varactor element attached to the Ag/AgCl electrode. This change is caused by the change in Cl^−^ concentration. Using near-field coupling, the resonance frequency is reflected on an antenna. The communication does not need battery/external power. The authors were able to reliably measure the Cl^−^ concentrations (0.01 to 0.2 M) up to 35 mm between readout and sensor coil.

Zhou et al. [39] proposed a wireless chloride sensor to monitor Cl^−^ concentration in concrete. The sensor is based on radio-frequency identification (RFID) communication protocol, which comprises an energy harvesting and management circuit, low-dropout (LDO) voltage regulator, microcontroller unit (MCU), RFID tag chip and three pairs of electrodes. These are: (i) calomel reference electrode, (ii) platinum, Pt, reference electrode, and (iii) Ag/AgCl electrode. The sensor tag powers its circuitry by harvesting energy which is radiated by the RFID reader. The authors also adopted a wavelet denoising approach to filter out the noise from the raw data which are corrupted by the noises in the wireless channel. In addition, monitoring software to display real-time measurements was developed. The performance of the sensor was tested in concrete with different Cl^−^ concentration. The authors claimed that the sensitivity of the developed wireless Cl^−^ sensor is high and the response time of the electrodes are sufficiently fast. The sensor reliably measures the Cl^−^ content in concrete within a communication distance of 16.3 m.

There are also studies which demonstrate the possibilities of integrating potentiometric sensors to monitor different parameters in a concrete environment. For instance, Duffó and Farina [40] developed integrated multifunctional sensors which monitor Cl^−^ content in concrete as well as open circuit potential and corrosion current density of rebar, electrical resistivity of concrete, oxygen availability, and inner temperature. The Cl^−^ sensor was developed based on Ag/AgCl electrodes. The evaluation of the sensors, in solutions of different pH values, exhibited acceptable stability and great reproducibility. The sensors have also demonstrated reasonably good stability when embedded in mortar for two years.

#### 2.1.2. Potentiometric pH Sensors

**Figure 3:**
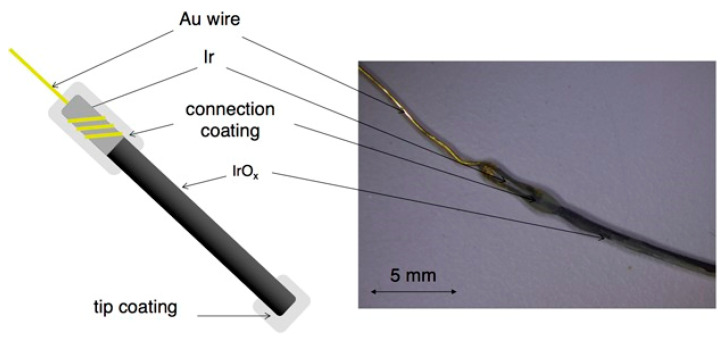
Schematic illustration and stereomicroscopy image of pH sensor based on an IrO_x_ electrode [50].

Monitoring the pH of the concrete is vital since the alkalinity of the concrete has paramount importance in protecting the embedded rebar against corrosion. Though the use of potentiometric sensors for pH measurement is a common practice in various application areas, its use in monitoring the pH of the concrete pore solution is very limited. It can be noticed from Table 3 that Ir/IrO_2_, Ag/Ag_2_O, Ti/IrO_2_, W/WO_3_, and MO electrodes are used in recent time to monitor pH in a concrete environment. Femenias et al. [49] utilized embeddable potentiometric pH sensor based on thermally-oxidized iridium/iridium oxide (IrO_x_) electrodes. They continuously monitor pH at different depths over time in mortar specimens subjected to accelerated carbonation. The authors claimed that the utilized pH sensors can provide insight into the carbonation process and in the kinetic processes, for example, transport and phases transformations. These authors, in another work [50], examined the feasibility of IrO_x_ electrodes for long-term continuous pH monitoring in a concrete environment, focusing on its reproducibility, stability, accuracy, and oxygen dependency. The sensor was exposed to highly alkaline solutions (pH 9–13.5) for about two years and they were able to measure with a maximum error of 0.5 pH units. They also examined its performance by embedding in mortar for about 160 days. The authors concluded that IrO_x_ electrodes-based sensors are stable, accurate, oxygen independent, and have outstanding ability for pH monitoring in a concrete environment. The formed (10–25 μm) thickness of the oxide layer is beneficial for long-term stability in concrete structure. Indeed, the authors indicated the essentiality of the IrO_x_ electrodes conditioning in highly alkaline solutions for a minimum of 3–4 months in order to achieve precise and reproducible potential responses. The response time of the electrodes are adequately fast for pH monitoring in a concrete environment. The schematic illustration and stereomicroscopy image of an IrO_x_ electrode that is developed to monitor pH in a concrete environment is shown in Figure 3.

Kolar et al. [51] examined the potentiometric response and stability of a W/WO_3_-based chloride sensor by exposing it to saturated calcium hydroxide solution for 10 months. The sensitivity was slightly decreased within the pH range of 5–12, but the response was stable and repeatable in high-alkaline solutions (pH > 12). The sub-Nernstian response was observed within the pH range of 2–5. None of the analysed interfering ions, SO_4_^2−^, K^+^, and Cl^−^, had a substantial impact on electrode potential. The W/WO_3_ electrode is robust, simple, low cost, and temperature resistant. By considering all these facts, the author reported that W/WO_3_ electrode is applicable for the long-term monitoring of Cl^−^ in concrete environment.

Similar to chloride sensors, Gandía-Romero et al. [52] developed a screen-printed Ag/Ag_2_O potentiometric pH sensor using thick-film technology and examined the performance of the sensor in simulated concrete pore solutions and concrete specimens. The performance evaluation of the sensor confirmed its reproducibility, reversibility, and acceptable response time. Based on these facts, the authors concluded that the screen-printed Ag/Ag_2_O sensors can be embedded into concrete to continuously monitor pH in real time, which in turn provide insight regarding the carbonation front.

Bhadra et al. [53] reported the development of an integrated wireless passive sensor which can monitor pH and temperature simultaneously in a concrete environment. This sensor comprises a pH electrode (Ir/IrO_x_), and a reference electrode (Ag/AgCl), a thermistor, a voltage sensing circuit and an interrogator coil which is inductively connected to the inductor of the sensor. The sensor was calibrated with a range of 25–55 °C and 1.5–12 for temperature and pH, respectively. By utilizing temperature compensation, a sensitivity of less than 0.1 pH was achieved with a response time of below 1 s. Nonetheless, this sensor is unstable to interference by the existence of other ionic matters. The authors claimed that the temperature compensation capacity and the design simplicity of the sensor make it suitable to be integrated by printed technology, so that the sensor will be used to remotely monitor pH in a concrete environment [53]. 

#### 2.1.3. Potentiometric Integrated Chloride Ion and pH Sensors

Monitoring of Cl^−^ concentrations and pH at rebar/concrete interface is imperative for detecting rebar corrosion at early stage and to make sound decisions. In this regard, there are some attempts which show the possibility of integrating chloride and pH sensors to monitor both parameters (Cl^−^ concentrations and pH values) simultaneously. For example, Du et al. [54] developed and presented an integrated sensor unit that monitor pH values and Cl^−^ concentration at the interface of rebar and concrete. The pH and the chloride sensors are based on Ir/IrO_2_ and Ag/AgCl electrodes, respectively. The sensor was embedded in the concrete to simultaneously monitor pH of the pore solution and the amount of Cl^−^ concentrations. The reported test results revealed that the integrated sensor is stable and robust, indicating the potential of the sensor to realize continuous in-service monitoring of pH and Cl^−^ concentrations in concrete. 

**Figure 4:**
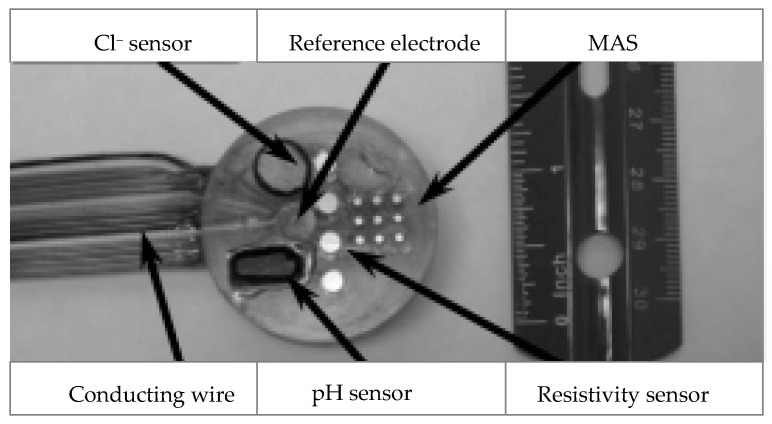
Image of the sensor package configuration, adopted from [55].

There are also pH and Cl^−^ sensors, which are integrated with other sensors that monitor different parameters in concrete environment. For example, Yu and Caseres [55] developed an embeddable prototype of an integrated multifunctional sensor unit. It encompasses electrodes of Ag/AgCl, metallic oxide (MO), a multi-electrode array sensor (MAS), and a four-pin (Wenner) array stainless steel for monitoring of Cl^−^ concentrations, pH, microcell corrosion current, and localized concrete resistivity, respectively. The long-term sensitivity and reliability of the prototype sensor was evaluated by embedding it in a cement paste cylinder. The claimed results (based on a year of monitoring) indicated that the pH and the other integrated sensors were reliable and stable, showing its potential to be implemented in real RC structures. Figure 4 illustrates the proposed multifunctional sensor unit. Dong et al. [56] also developed multifunctional sensor to monitor pH of the pore solution, Cl^−^ concentration in concrete, corrosion current, and equilibrium potential of rebar. The pH sensor was developed from Ti/IrO_2_ electrode, while the Cl^−^ sensor from Ag/AgCl electrode. The reported results of the investigation demonstrated that all the sensors (including pH and Cl^−^) were stable and reliable while providing great potential responses. Hence, this multifunctional sensor is suitable for long-term in-service monitoring of corrosion causing parameters. 

### 2.2. Fiber-Optic Sensors

As ISEs measurement, optical techniques are one of the oldest and well-established methods for detecting chemical analytes and have shaped the foundation for several chemical sensors. The optical based sensing technology is not based on a single concept but on a variety of optical phenomena that can be applied to measure a wide range of chemical and physical parameters [57,58]. The evolution of low-cost high-quality optical fibers has facilitated the wide development and adoption of optical fiber sensors. A fiber-optic sensor (FOS), in its simplest form, comprises optical fiber, light source, sensing element, and detector. The fundamental operation principle of fiber-optic sensors is that the sensing element modulates one or more parameters of the optical system (e.g., wavelength, chromatic, phase, polarization, etc.), which results in a change in the optical signal characteristic at the detector. FOS can influence the light-guiding features of the transmitting fiber and correlate the specific attributes to the changes of the light. The basic principle is to evaluate an external physical parameter by causing changes in the optical characteristics of a light beam passing inside and along an optical fiber. The fiber is therefore both the transmission medium and the sensing element [58]. 

In the past four decades, there has been substantial research attempt in developing optical fiber- based sensors for both chemical and physical analytes. Accordingly, several interesting schemes have been made and continue to be the subject of significant research. Application areas for chemical analysis involve laboratory-based analysis, environmental monitoring, and clinical diagnosis applications. Every area of specific application has their own prerequisites for selectivity, sensitivity, accuracy, robustness, and cost. The studied physical state of the analytes comprised gases, dissolved gases, liquids, ions in solution, and solids [57]. With FOS, chemical analysis can often be carried out in situ and in real time using various approaches, which are: fluorescence, scattering, colour change, absorption, evanescent wave interaction, and refractive-index change [58]. Fiber-optic fluorescence techniques have been used to measure various types of ions, such as chloride, iodide, iron, plutonium, and sulphate. It has also been expanded to measure pH. 

#### 2.2.1. Fiber-Optic Chloride Ion Sensors

**Figure 5:**
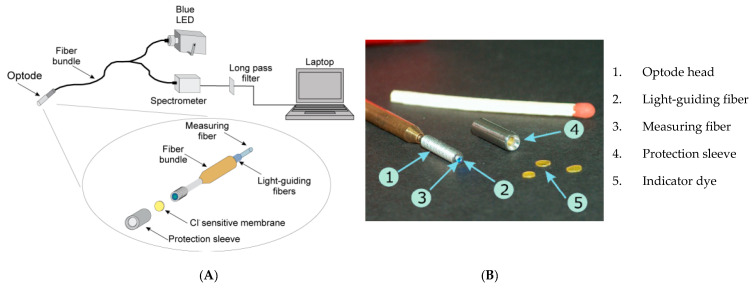
(**A**) Schematic representation of optical fiber based Cl^−^ sensor measurement setup, (**B**) components of the sensor [60].

Recently, few numbers of fiber optic-based sensors to monitor Cl^−^ concentrations in concrete environment was developed. Ding et al. [59] have developed Cl^−^ sensor using a suspended-core optical fiber for in-situ real-time monitoring. The predominant property of suspended-core optical fiber is its big air holes running throughout its length, and thus sensitive material could be stored into the holes. This type of optical fiber has extended interaction length, powerful light-matter interaction and utilization of small sample amounts [59]. They introduced a sol-gel membrane to immobilize lucigenin (used as a fluorescence sensitive material) over the internal wall of the suspended-core optical fiber using dip-coating technique. The performance of the sensor with interfering ions (Na^+^, Ca^2+^, Mg^2+^, SO_4_^2−^, and OH^−^) that can be occurred in concrete was tested. The authors claimed that the sensor works in high-alkaline environments. Na^+^, Ca^2+^, and Mg^2+^ have minor influence on Cl^−^ detection of the fiber-optics sensor and high amount of SO_4_^2−^ slightly hamper the process of lucigenin quenching. The OH^−^ has a major interference in Cl^−^ detection of the sensor. Based on the results, the authors claimed that the developed sensor could be successfully applied for Cl^−^ monitoring in concrete structure. Laferrière et al. [60] has developed optical fiber for real-time and continuous monitoring of Cl^−^ concentrations in concrete pores. It uses Cl^−^ sensitive fluorescence indicator dye. The performance of the developed sensor was tested in mortar specimens at various depths in two simulated climatic conditions: (i) hot maritime and (ii) cold environment. The authors reported that it is possible to precisely quantify the Cl^−^ concentrations in both climatic conditions, indicating its applicability for scientific studies and condition monitoring of concrete structure. The schematic representation of the optical fiber based Cl^−^ sensor measurement setup and the components of the sensor is illustrated in Figure 5.

#### 2.2.2. Fiber-Optic pH Sensors

Though fiber-optic fluorescence techniques are applied to measure pH in various fields of study, its application in concrete environments is yet limited. Nguyen et al. [3,61] produced an optical fiber sensor using pH sensitive fluorescent polymer to monitor pH of concrete pores. The authors stated that the sensor provides responses over the appropriate pH range for concrete with an acceptable rate of around 50 min and is stable over a period of 20 months. They also reported that the sensor has trivial cross sensitivity to ionic strength and outstanding photostability in a series of tests. All these characteristics make the sensor suitable for long-term in-service monitoring of pH in concrete. McPollin et al. [62] prepared tailor-made fiber-optic sol-gel-based pH sensors aiming to have a more suitable characteristic in concrete environments. It was produced by applying the sol-gel coating (manufactured from tetraethyl orthosilicate (TEOS)) onto a plastic clad silica fiber with a core diameter of 600 μm. The adopted indicator dye was cresol red, which has pH range from 8–13. Hydrochloric acid (HCl) was applied as a catalyst solution. The performance of the sensor was assessed by embedding it in cement mortar cubes for more than 18 months and it was functional through the testing period.

### 2.3. Comparison between Potentiometric and Fiber-Optic Sensors

As discussed earlier, the operational technique of the potentiometric and fiber-optic sensors is different. Since both types of sensing devices are based on their own specific principles; they are affected by different parameters. Thus, the comparison between them is focused on their production process, invasiveness, and performance. 

The production process of potentiometric sensors is simple which adopts the well-known electrochemical processes, whereas the fiber-optic sensors are highly technical and complicated. Regarding invasiveness, both types of sensing devices have negligible effect on the structural behaviour of the infrastructure because they are small compared with the size of the structural element under investigation. They also have no impact on the surrounding environment during measuring Cl^−^ and pH in the concrete element. 

The overall performance of the potentiometric sensors largely depends on the types and preparation of the electrodes. As several of the parameters (such as the types of electrodes, the production processes of the sensors, the test environment, and test duration of the sensors) presented in Table 3 are varying considerably, it is impossible to compare the performance of the sensors, rather providing the general observations. Generally, all the potentiometric sensors presented in Section 2 are sufficiently accurate and stable based on the measurements taken up to the period of two years. Both the potentiometric as well as the fiber-optic sensors can be affected by the presence of some interfering ions. However, more types of ions can interfere with the potentiometric based sensing devices than FOS. The fiber-optic sensors are found to be more chemically stable than potentiometric sensors.

## 3. Internet of Things Based Corrosion Monitoring

The Cl^−^ and pH sensors presented above can be embedded in real RC structures for corrosion monitoring as almost all the authors concluded that both potentiometric and fiber-optic sensors are reasonably precise, stable, and reliable. This allows avoiding the conventional laboratory based destructive testing and paves a way for continuous monitoring. All but three of the sensors that are presented in Section 2 transfer their measurement to a data logger through wired connection. Deployment of such kind of wired sensors in spatially dispersed concrete elements of a particular structure bears substantial costs for installing and retaining the wired system. It is also inflexible and unattractive aesthetically. Three of the sensors [39,48,53] that employ wireless communication for transmitting their measurements use very short-range communication technology that require close-range reading. This limits their applicability for continuous long-term monitoring. The above reviewed sensors were tested for up to two years, which is very short compared to the service life of RC structures that often spans about 50–80 years. This requires reliable operation of sensors for a long period. This could be achieved through the integration of self-diagnosis and self-repair capability in the sensing subsystems and/or possibility to easily replace them without affecting the monitoring system. It is also necessary to have energy scavenging capability for ensuring the availability of energy sources despite depleted battery sources. In the area of structures, the commonly employed energy scavenging method is converting vibrations into electrical energy. A review of energy harvesters that converts vibrations into electrical energy can be found in [63].

**Figure 6:**
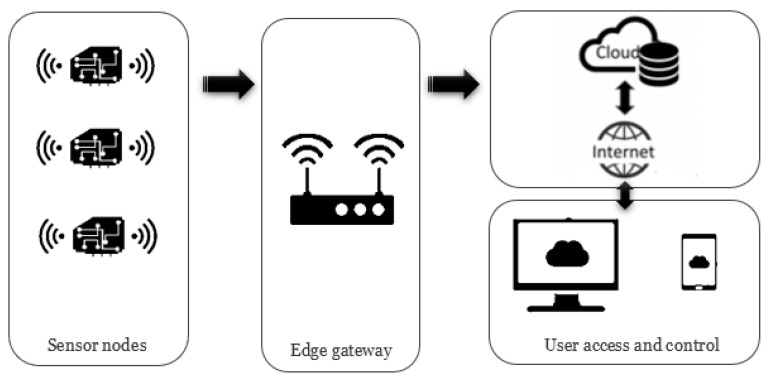
Schematic representation of the main components of IoT.

The IoT is a networked system where distributed sensors monitor physical events and transmit the measurements through wireless communication medium to a remote user. IoTs have been proposed for a number of different applications, such as healthcare [64,65,66], environmental monitoring [67,68,69], smart home and city [70,71,72], and structural health monitoring [73,74,75]. The main components of IoT are sensor nodes (comprising sensors, low-power computing devices, and radio modules for wireless communication), gateway/edge devices, and cloud as illustrated in Figure 6. Several low-power and low-complexity computing devices are developed for IoT applications, which are usually battery powered. The sensors are integrated to the computing device through wired connection. The computing device performs signal processing operations on the sensor’s measurement according to the application requirements and prepares it for transmission by the integrated/interconnected radio module. 

Various low-power communication technologies are developed for transmitting the data from the computing device to the gateway. These technologies differ in their communication range, spectrum usage, bandwidth requirement and power consumption. For corrosion monitoring of RC structures, communication technologies that have 100 m or longer communication range, such as Zigbee [76], LoRa [77], Sigfox [77], and narrowband IoT [77], are better choices. The long-range communication technologies, such as LoRa, Sigfox, and narrowband IoT enable wide area networks mainly using a star topology where the measurement is directly communicated to the gateway without the need for a relay node. These technologies are a better option for monitoring large RC structures in a cost-effective manner as it requires a smaller number of nodes. LoRa and Sigfox operate in the unlicensed spectrum where Sigfox requires some form of subscription fee. Narrowband IoT uses licensed spectrum, which needs payment for the service to a mobile service provider. The gateway is connected on one side to the IoT’s low-power computing nodes through low-power wireless communication technologies and on the other side to the edge device/cloud through the Internet. The gateway translates the communication protocol and forwards the data to the edge device/cloud. The collected data in the edge device/cloud is processed and communicated with the remote user through appropriate web applications.

IoT-based corrosion monitoring system has advantages over wired sensors with datalogger systems in terms of continuous measurements, cost effectiveness, flexibility, remote monitoring, ease of deployment and maintenance. There have been proposals on the use of wireless sensor networks for corrosion detection [78,79,80,81] and IoT for structural health monitoring [73,74,75]. Among these works, Qiao et al. [79], Sun et al. [80], and Xu et al. [81] employed IEEE802.15.4-based short-range connectivity for corrosion monitoring while Abdelgawad and Yelamarthi [74] used WiFi connectivity for structural health monitoring. The rest of the works have failed to mention the specific connectivity technology that is considered in their work. The choice on short- vs. long-range connectivity technology depends on the size of the structure and aesthetic importance besides specific performance requirements of the communication technology. IoT nodes can be deployed on any type of RC structures and deliver facility managers/owners with indispensable real-time data on the corrosion condition of the structure, and thus making informed decisions.

## 4. Evolution of Data-Driven Corrosion Assessment

With the emerging IoT based low-cost continuous monitoring system, speculating about where the future might lead in corrosion assessment of RC structures is interesting. It is well known that smart environments based on IoTs and complex data analytical techniques represent the next evolutionary development step in various disciplines, including transport [82], military [83], agriculture [84,85], and civil engineering [30]. The complex data analytics is often performed using machine learning and/or deep learning methods. Machine learning is a subdomain of artificial intelligence that engages design and implementation of algorithms to recognize intricate patterns out of data and make intelligent decisions [86,87,88,89,90,91,92,93,94]. It models profound relationships in data inputs and reconstructs a knowledge scheme. Machine learning based models can be predictive to carryout prediction or descriptive to discover knowledge from data, or both without assuming a predetermined equation as a model [90,95,96]. It is one of the foremost outstanding technological developments in recent history and used in various disciplines, e.g., computational finance [97,98,99], image and speech processing [100,101,102], property valuation [103,104,105], computational biology [106,107,108], and energy production [109,110,111]. Deep learning is a subset of machine learning that uses multiple layers to progressively extract features from the raw input data [112]. Both, machine learning and deep learning, have strong abilities to handle heterogeneous and large volume of data and achieve high accuracy and reliability, which make them suitable for making a correct decision for various complex problems. Even if employing intelligent data analytics is already becoming a common practice in different disciplines, since recent times, its use in the field of concrete durability is at its infancy. For example to detect rebar corrosion [79,81,113], to characterize carbonation resistance [114,115,116,117,118,119,120], Cl^−^ permeability [121,122,123,124,125,126], hygrothermal performance in concrete [127,128], Cl^−^ diffusion coefficient [129,130,131,132], chloride penetration and carbonation predictors [116,133], and. Indeed, these works largely employ short-term data generated either from accelerated laboratory tests or field tests, but not real-time data from embedded sensors. A comprehensive review regarding the application of machine learning in the field of concrete durability is found in [134].

Continuous in-situ monitoring of parameters that initiate and accelerate rebar corrosion in concrete structure is crucial to avoid corrosion associated damage. An early detection of corrosion risks assists engineers to plan optimal maintenance measures in a timely and cost-effective manner. Long-term in-situ monitoring of such parameters has also the utmost importance in better understanding of the complex corrosion phenomenon. With the current development of sensing technology, realization of miniaturized sensors, that are stable, accurate, robust, and low-cost, for monitoring durability performance of RC structures will grow in years to come. These sensors can be employed in RC structures to monitor factors that control corrosion of rebar or other deterioration mechanisms, even in remote locations that are normally inconvenient to access. Data acquired from several sensors will be fused using machine learning and/or deep learning methods. The fused data will then be used to evaluate the status of the structures autonomously by applying appropriate algorithm(s). Several machine learning algorithms such as k-nearest neighbors, linear models, decision trees, ensembles of decision trees, and support vector machine as well as deep learning algorithms such as convolutional neural network, recurrent neural networks, long short-term memory networks and deep belief networks, have the potential to be employed for realizing autonomous corrosion assessment method. Thus, the application of IoT systems and intelligent data analytics will form a principal element in lifecycle management of RC structures.

**Figure 7:**
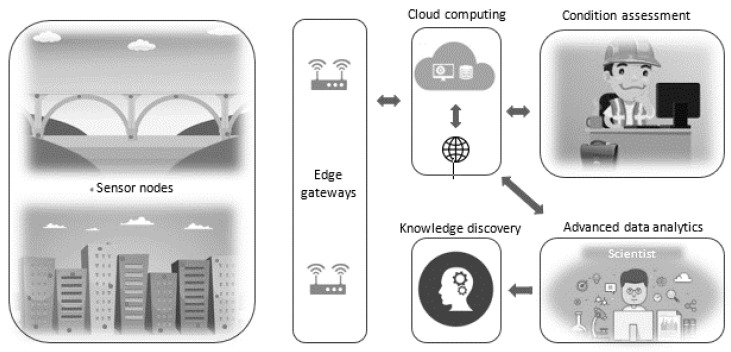
Schematic presentation of autonomous corrosion assessment.

Autonomous corrosion assessment utilizing IoT systems and advanced data analytics is illustrated in Figure 7. As can be seen in the figure, chloride and pH sensors are embedded in RC structures along with other relevant sensors such as moisture and temperature sensors. As it is described earlier, moisture and temperature affect the critical chloride threshold. These environmental loads are also the major factor that are controlling the corrosion rate of rebar. The moisture amount within the concrete governs the corrosion rate through their influence on the electrochemical reactions at the rebar-concrete interface and ions transport between anodes and cathodes [12]. The surrounding temperature controls the rate of rebar corrosion since it influences the electrochemical reactions and the moisture amount that the concrete retains [135]. For example, the rate of corrosion differs by more than a factor of ten in a regular seasonal temperature range of 5 to 30 °C [6,135,136]. Thus, continuous hygrothermal monitoring of concrete is necessary. This provides more reliable information about the actual hygrothermal behavior of the concrete. Today, there are several studies which show the applicability of wireless sensors for monitoring moisture and temperature in concrete environments [81,137,138,139]. Continuous monitoring of such parameters using sensing devices will deliver more reliable data compared to carrying out periodic field testing. In the long term, this approach leads to cost savings, considering the users cost, the labour cost, and their safety into account. This is because such a monitoring system delivers continuous in-service data without the involvement of inspection crews on the field. By applying machine learning and or deep learning on the collected data, accurate corrosion assessment can be computed in the cloud autonomously and communicated to remote stakeholders. This allows the responsible parties to carry out condition-based maintenance actions in a timely manner, and thus alleviates maintenance-associated costs substantially. Such autonomous corrosion condition assessment methods, which add a digital layer to the physical infrastructure, make the infrastructure smart. Scientists and other interested parties can make use of the data available in the cloud to carry out in-depth studies, which enable them to generate new knowledge that help to address corrosion associated damages. The uncovered scientific knowledge will support material engineers to design robust concrete solutions that enhance the durability of the infrastructure. In addition, the long-term data and its analysis assist engineers to devise optimal maintenance plans.

## 5. Conclusions

Continuous monitoring of corrosion-causing factors in a reliable and cost-effective manner is vital to plan accurate condition-based maintenance strategies for RC structures. In this paper, state-of-the-art pH and Cl^−^ sensors that are proposed for monitoring the carbonation front and Cl^−^ concentration in concrete environment are critically reviewed. Based on their measuring principles, the sensors utilized in concrete environment for monitoring pH and Cl^−^ concentration can be categorized into two: potentiometric and fiber optic. The majority of the studied pH and Cl^−^ sensors have showed high sensitivity, reliability, and stability in concrete environment, though the experiments were carried out for relatively short periods. The review work also identified three cases that attempted to monitor the Cl^−^ in concrete wirelessly albeit very short range. In addition, this work speculates where the future might lead in corrosion condition assessment of RC structures. With the technology development of sensors along with IoT-based systems, and machine learning, autonomous corrosion condition assessment remotely in a cost-efficient manner will become feasible. The collected sensor data can be analysed further by engineers and scientists for greater understanding concerning the intricate corrosion phenomena. The new scientific knowledge will help scientists to design optimum solutions that increase the durability of infrastructure and to build proactive maintenance plans.

## Figures and Tables

**Table 1:** Search details and results.

Search Terms	Selection Criteria	Search Result					Total Number of Studies Included
		Document Type	Scopus		Web of Science		
			Open Access	Closed Access	Open Access	Closed Access	
Chloride sensor, concrete	10 years (2009–2019)	Original article	6	6	4	8	21
		Conference paper	-	4	-	-	
		Book chapter	-	1	-	-	
		Conference review	-	1	-	-	
		Review article	1	-	1	-	
pH sensor, concrete	10 years (2009–2019)	Original article	2	8	5	3	13
		Conference paper	-	3	-	1	
		Book chapter	-	3	-	-	
		Conference review	-	-	-	-	
		Review article	1	1	-	1	
Total			10	27	10	13	34

**Table 2:** Characteristics of commonly applied reference electrodes with their potentials versus NHE, adopted from [34].

Electrode	Type	Potential (mV vs. NHE)	Typical Configuration
Calomel	Unpolarizable	+244	Mercury chloride (Hg_2_Cl_2_) paste on mercury (Hg) and a base metal rod (Pt) in a solution of potassium chloride (KCl).
Copper/copper sulphate		+316	Copper rod surrounded by a saturated solution of copper sulphate.
Silver/silver chloride		+199	Silver rod in silver chloride paste in a saturated solution or 0.5 M KCl solution.
Manganese dioxide		+365	Manganese dioxide paste on a base material (graphite) in 0.5 M potassium hydroxide (KOH) solution with a cementitious plug as contact to the concrete.
Graphite	Polarizable	+150 ± 20	Delivered with an isolating jacket leaving the tip exposed.
Activated titanium		+150 ± 20	
Stainless steel		+150 ± 20	
Lead		−450	

**Table 3:** State-of-the-art Cl^−^ and pH potentiometric sensors details, test environment and their performance.

No.	Sensor Types	Electrode Used	Tested Environment	The Examination Focuses on	Exposure Time	Performance Evaluation	Publication Year	Data Transmission Method	Reference
1	Cl^−^	Ag/AgCl	simulated concrete pore solution	- neutral solution - alkaline solution - interfering ions	2 months	The sensor exhibits insignificant interference from fluoride, sulphate, and hydroxyl however substantial from bromide and sulphide. In completely chloride-free alkaline solutions, the ISEs were not stable over time, but upon arrival of Cl^−^, it reliably measures the Cl^−^ concentrations.	2016	wired	[44]
2		Ag/AgCl	mortar	- wide range of Cl^−^ concentrations - different depths	20 days	The sensor enabled determination of Cl^−^ concentrations in mortar specimens that nearly followed Fick’s law for transient diffusion. In concrete it is found to be reasonably stable for the duration of the experiment. It also exhibits good sensitivity in a wide range of Cl^−^ concentrations.	2006	wired	[38]
			concrete		~100 days				
3		Ag/AgCl	simulated concrete pore solution	- wide range of Cl^−^ concentrations - different pH values - different solutions	1000 s	Cl^−^ concentration at 1000 mM, the composition of the solution has a minimum effect on the sensor’s response. The influence of pH on the potential value of the sensor is trivial at Cl^−^ concentrations of >4 mM, and thus the pH value must be simultaneously monitored to accurately determine the Cl^−^ content with minimal concentration.	2014	wired	[41]
4		Ag/AgCl	simulated concrete pore solution	- neutral solution - alkaline solution	>6 months	The sensor was affected by the pH of the solution in the complete absence of Cl^−^, but in the presence of Cl^−^ it showed good long-term stability even in high-alkaline solutions.	2010	wired	[32]
			mortar		~2 months				
5		Ag/AgCl	concrete	- various concrete types - different depths	2 years	Based on the experiments carried out in concrete specimens composed of different mineral admixtures, the authors conclude that continuous monitoring of Cl^−^ concentration in concrete structure could be achieved. They also remarked that extra efforts are needed to develop low-cost, long-term, robust, and reliable Cl^−^ potentiometric sensors to attain extensive applications in concrete environment.	2017	wired	[43]
6		thick-film Ag/AgCl	simulate concrete pore solution	- different depths - wide range of Cl^−^ concentrations - water content of concrete - different pH values	Not stated	The sensors respond to the activity of free Cl^−^ in the concrete pore solution. The electrical potential of the sensor relies on the water content of the concrete. The thickness gained from the composition of the thick-film technique (10 μm) and resistive pastes enhances its durability. It is a promising Cl^−^ sensor for concrete structure since they are robust, miniaturized, inexpensive, and have long-term stability.	2016	wired	[42]
			concrete		~62 days Not clearly stated, deduced from results				
7		polymer coated Ag/AgCl	concrete	- wide range of Cl^−^ concentrations - different depths - different pH values	60 days	The sensor exhibited outstanding chloride sensing ability. It is well stable in an alkaline medium. The existence of the coating polymer prevented the formation of Ag_2_O in the electrode.	2017	wired	[45]
8		MnO_2_ Ag/AgCl	simulated concrete pore solution	- wide range of Cl^−^ concentrations - interfering ions - different pH values - response against temperature	90 days	The sensor is slightly influenced by the interfering ions of K^+^, Ca^2+^, Na^+^, and SO_4_^2−^, but considerably affected by the pH at low chloride concentration. Over the range from 5 to 45 °C, the sensor’s potential reading linearly grows with the solution temperature and has excellent polarization behaviour.	2010	wired	[46]
9		Ag/AgCl	simulated concrete pore solution	- wide range of Cl^−^ concentrations - interfering ions	3 months	It reveals acceptable sensitivity to Cl^−^ and clear Nernstian relationship between potential response and wide range of Cl^−^ concentration. There is insignificant discrepancy of electrode’s potential response due to the interfering ions of K^+^, Ca^2+^, Na^+^, and SO_4_^2−^.	2011	wired	[47]
10		Ag/AgCl	concrete	- wide range of Cl^−^ concentrations - capacitance measure - communication distance	Not stated	Reliable capacitance measurement, which is caused by the change in Cl^−^ concentration from 0.01 to 0.2 M. The measurements are reliable up to 35 mm between sensor and readout coil. The communication does not need battery/external power.	2015	wireless	[48]
11		Ag/AgCl	concrete	- wide range of Cl^−^ concentrations - communication distance	15 days	The sensitivity of the sensor to Cl^−^ is high and the response time of the electrodes are sufficiently fast. It reliably measures the Cl^−^ content in concrete within a communication distance of 16.3 m.	2017	wireless	[39]
12 ^††^		Ag/AgCl	simulated concrete pore solution	- different pH values	2 years	The sensor exhibited acceptable stability and great reproducibility in simulated concrete pore solution and other liquid solutions of different pH values. The sensors embedded in the mortar also demonstrated reasonably good stability.	2009	wired	[40]
			mortar						
13	pH	Ir/IrO_2_	mortar	- wide range of depths	~160 days	The authors utilized an embeddable pH sensor based on thermally oxidized Ir/IrO_2_. The results from the sensor provide insight in the carbonation process and in the kinetic processes, such as transport and phases transformations.	2017	wired	[49]
14		IrO_x_	alkaline test solutions	- potential pH-response - reproducibility - accuracy - oxygen dependency	~2 years	The sensor is able to measure the pH with a maximum error of 0.5 units in a pH range of 9–13.5. It is stable, oxygen independent, and delivers precise and reproducible potential-pH responses. However, the electrode requires conditioning in highly alkaline solutions for at a minimum of 3–4 months. The formed (10–25 μm) thickness of the oxide layer is beneficial for long-term stability in concrete structure.	2017	wired	[50]
			mortar		160 days				
15		W/WO_3_	simulated concrete pore solution	- different pH values - interfering ions	10 months	The sensitivity was slightly decreased within the range from pH 5 to 12, but the responses are stable and repeatable to alkaline solutions (pH > 12). The sub-Nernstian response was observed within the range from pH 2 to 5. All the analysed interfering ions, SO_4_^2−^, K^+^, and Cl^−^, had no substantial impact on electrode potential. The electrode is robust, simple, low cost, and temperature resistant.	2010	wired	[51]
16 ^†††^		thick-film Ag/Ag_2_O	simulated concrete pore solution	- to characterize the sensor	time varies based on the property under investigation	Ag_2_O electrodes exhibited excellent electrochemical response to pH variations in the solution. Indeed, electrode potential variation was observed when the Cl^−^ concentration is about 10−2.5 M. With the rising of temperature, the average experimental slope slightly increases like the theoretical ones. In general, it reveals very good reproducibility, reversibility, and an acceptable response time. The sensor array allows the authors to monitor the carbonation progress in hardened concrete.	2016	wired	[52]
			concrete	- interfering ions - wide range of depths - response against temperature	19 days				
17 ^†^		Ir/IrO_x_ Ag/AgCl	solutions of different pH	- response against temperature - different pH values - Interrogator-sensor separation distance	2 days	By utilizing temperature compensation, a sensitivity of less than 0.1 pH was achieved with a response time of below 1 s. A resonant frequency change less than 8 kHz and a quality factor variation of 1.32 were obtained with separation distances between 2.5 and 8.5 cm. The temperature compensation ability and the design simplicity of the sensor make it suitable to be integrated by printed technology.	2013	wireless	[53]
18 ^†^	pH/Cl^−^	Ir/IrO_2_	simulated concrete pore solution	- different pH values - wide range of Cl^−^ concentrations	100 days	The integrated pH/Cl^−^ sensor exhibited good linear responses to the logarithm of the Cl^−^ concentration (1 × 10^−4^–2 M) and pH 1–14. It is stable, robust, and sensitive, indicating its potential to realize in situ and long-term monitoring of pH values and Cl^−^ concentrations in concrete environment.	2006	wired	[54]
		Ag/AgCl							
19 ^††^		MO	cement paste	- different pH values - wide range of Cl^−^ concentrations	1 year	The pH/Cl^−^ probes were calibrated in simulated pore solutions concerning temperature and pH fluctuations. After calibration, it was tested in cement paste. The result demonstrated that the sensor is reliable and stable.	2012	wired	[55]
		Ag/AgCl							
20 ^††^		Ti/IrO_2_	concrete	- different pH values - wide range of Cl^−^ concentrations	224 days	The pH/Cl^−^ probes have great sensitivity, reliability, and potential responses in a wide range of pH and Cl^−^ concentrations. This multifunctional sensor is also used to monitor the corrosion behaviour of rebar in concrete.	2011	wired	[56]
		Ag/AgCl							

^†^ two integrated sensors, ^††^ more than two integrated sensors, ^†††^ manufactured using screen-printed technology.

## References

[1] World Bank (2017). Gross Domestic Product 2016.

[2] Hoult N., Bennett P.J., Stoianov I., Fidler P., Maksimović Č., Middleton C., Graham N., Soga K. (2009). Wireless sensor networks: Creating “smart infrastructure”. Proc. Inst. Civ. Eng. Civ. Eng..

[3] Nguyen T.H., Venugopala T., Chen S., Sun T., Grattan K.T.V., Taylor S.E., Basheer P.A.M., Long A.E. yFibre optic pH sensor for Corrosion Monitoring in Concrete Structures. Proceedings of the 14th International Meeting on Chemical Sensors—IMCS 2012.

[4] ASCE (2017). 2017 Infrastructure Report Card: A Comprehensive Assessment of America’s Infrastructure.

[5] Yu B., Yang L., Wu M., Li B. (2014). Practical model for predicting corrosion rate of steel reinforcement in concrete structures. Constr. Build. Mater..

[6] El-Reedy M. (2008). Steel-Reinforced Concrete Structures: Assessment and Repair of Corrosion.

[7] Zhou Y., Gencturk B., Willam K., Attar A. (2015). Carbonation-induced and chloride-induced corrosion in reinforced concrete structures. Mater. Civ. Eng..

[8] Singh R. (2014). Corrosion Control for Offshore Structures: Cathodic Protection and High-Efficiency Coating.

[9] Matthews S.L., Morlidge J.R., Alexander M.G., Beushausen H.-D., Dehn F., Moyo P. (2008). Performance based rehabilitation of reinforced concrete structures. Concrete Repair, Rehabilitation and Retrofitting II: 2nd International Conference on Concrete Repair, Rehabilitation and Retrofitting, ICCRRR-2.

[10] Vaysburd A.M., Emmons P.H., Bissonnette B., Alexander M.G., Beushausen H.-D., Dehn F., Moyo P. (2008). Concrete repair: Research and practice—The critical dimension. Concrete Repair, Rehabilitation and Retrofitting II: 2nd International Conference on Concrete Repair, Rehabilitation and Retrofitting, ICCRRR-2.

[11] Taffese W.Z., Sistonen E. (2013). Service life prediction of repaired structures using concrete recasting method: State-of-the-art. Procedia Eng..

[12] Song D., Yang F., Guo M., Zhao S., Hao J., Chen Z., Sun J., Xu Y., Jiang J. (2019). Surface modification of rusted rebar and enhanced passivation/anticorrosion performance in simulated concrete pore solutions with different alkalinity. Metals.

[13] Neville A.M., Brooks J.J. (2010). Concrete Technology.

[14] Mehta P.K., Monteiro P.J.M. (2006). Concrete: Microstructure, Properties, and Materials.

[15] fib (International Federation for Structural Concrete) (2009). Structural Concrete: Textbook on Behaviour, Design and Performance.

[16] Lagerblad B. (2005). Carbon Dioxide Uptake during Concrete Life Cycle–State of the Art.

[17] Huang Q., Jiang Z., Zhang W., Gu X., Dou X. (2012). Numerical analysis of the effect of coarse aggregate distribution on concrete carbonation. Constr. Build. Mater..

[18] Ann K.Y., Pack S.W., Hwang J.P., Song H.W., Kim S.H. (2010). Service life prediction of a concrete bridge structure subjected to carbonation. Constr. Build. Mater..

[19] Wang X.-Y., Lee H.-S. (2009). A model for predicting the carbonation depth of concrete containing low-calcium fly ash. Constr. Build. Mater..

[20] Tang L., Nilsson L.-O., Basheer P.A.M. (2012). Resistance of Concrete to Chloride Ingress: Testing and Modelling.

[21] Nguyen T.S., Lorente S., Carcasses M. (2009). Effect of the environment temperature on the chloride diffusion through CEM-I and CEM-V mortars: An experimental study. Constr. Build. Mater..

[22] Ye H., Jin X., Fu C., Jin N., Xu Y., Huang T. (2016). Chloride penetration in concrete exposed to cyclic drying-wetting and carbonation. Constr. Build. Mater..

[23] Zhu X., Zi G., Cao Z., Cheng X. (2016). Combined effect of carbonation and chloride ingress in concrete. Constr. Build. Mater..

[24] Torres-Luque M., Bastidas-Arteaga E., Schoefs F., Sánchez-Silva M., Osma J.F. (2014). Non-destructive methods for measuring chloride ingress into concrete: State-of-the-art and future challenges. Constr. Build. Mater..

[25] Angst U., Elsener B., Larsen C.K., Vennesland Ø. (2009). Cement and concrete research critical chloride content in reinforced concrete—A review. Cem. Concr. Res..

[26] Ahlström J., Tidblad J., Sederholm B., Wadsö L. (2016). Influence of chloride and moisture content on steel rebar corrosion in concrete. Mater. Corros..

[27] Elsener B., Zimmermann L., Böhni H. (2003). Non destructive determination of the free chloride content in cement based materials. Mater. Corros..

[28] McCarter W., Chrisp T., Starrs G., Adamson A., Owens E., Basheer P., Nanukuttan S., Srinivasan S., Holmes N. (2012). Developments in performance monitoring of concrete exposed to extreme environments. Infrastruct. Syst..

[29] McCarter W., Chrisp T., Starrs G., Holmes N., Basheer L., Basheer M., Nanukuttan S. (2010). Developments in monitoring techniques for durability assessment of cover-zone concrete. Proceedings of the 2nd International Conference on Durability of Concrete Structures.

[30] Taffese W.Z. (2020). Data-driven method for enhanced corrosion assessment of reinforced concrete structures. arXiv.

[31] Dansk Standard (2004). Repair of Concrete Structures to EN 1504.

[32] Angst U., Elsener B., Larsen C.K., Vennesland Ø. (2010). Potentiometric determination of the chloride ion activity in cement based materials. J. Appl. Electrochem..

[33] Bagheri S., Amiri I.S., Yousefi A.T., Hamid S.B.A. (2017). Nanocomposites in Electrochemical Sensors.

[34] Vennesland Ø., Raupach M., Andrade C. (2007). Recommendation of Rilem TC 154-EMC: “Electrochemical techniques for measuring corrosion in concrete”—Measurements with embedded probes. Mater. Struct..

[35] McCarter W.J., Vennesland Ø. (2004). Sensor systems for use in reinforced concrete structures. Constr. Build. Mater..

[36] Bertolini L., Elsener B., Pedeferri P., Polder R. (2005). Corrosion of Steel in Concrete: Prevention, Diagnosis, Repair.

[37] De Brito P.S.D., Cunha P.T., Ferreira M.G.S. (2013). Solid potential reference electrode for concrete corrosion monitoring. Sensors Mater..

[38] Montemor M.F., Alves J.H., Simões A.M., Fernandes J.C.S., Lourenço Z., Costa A.J.S., Appleton A.J., Ferreira M.G.S. (2006). Multiprobe chloride sensor for in situ monitoring of reinforced concrete structures. Cem. Concr. Compos..

[39] Zhou S., Sheng W., Deng F., Wu X., Fu Z. (2017). A novel passive wireless sensing method for concrete chloride ion concentration monitoring. Sensors.

[40] Duffó G.S., Farina S.B. (2009). Development of an embeddable sensor to monitor the corrosion process of new and existing reinforced concrete structures. Constr. Build. Mater..

[41] Pargar F., Koleva D.A., Copuroglu O., Koenders E.A.B., van Breugel K., Mangabhai R.J., Bai Y., Goodier C.I. (2014). Evaluation of Ag/AgCl sensors for in-situ monitoring of freee chloride concentration in reinforced concrete structures. Young Researchers’ Forum II: Construction Materials.

[42] Gandía-Romero J.M., Bataller R., Monzón P., Campos I., García-Breijo E., Valcuende M., Soto J. (2016). Characterization of embeddable potentiometric thick-film sensors for monitoring chloride penetration in concrete. Sens. Actuators B Chem..

[43] Jin M., Jiang L., Zhu Q. (2017). Monitoring chloride ion penetration in concrete with different mineral admixtures based on embedded chloride ion selective electrodes. Constr. Build. Mater..

[44] Femenias Y.S., Angst U., Caruso F., Elsener B. (2016). Ag/AgCl ion-selective electrodes in neutral and alkaline environments containing interfering ions. Mater. Struct..

[45] Karthick S., Kwon S.-J., Lee H.S., Muralidharan S., Saraswathy V., Natarajan R. (2017). Fabrication and evaluation of a highly durable and reliable chloride monitoring sensor for civil infrastructure. RSC Adv..

[46] Gao X., Zhang J., Yang Y., Deng H. (2010). Fabrication and Performance of All-Solid-State Chloride Sensors in Synthetic Concrete Pore Solutions. Sensors.

[47] Gao X.J., Zhang J., Yang Y.Z., Lu S. (2011). Preparation of Chloride Ion Selective Electrode and its Potential Response to Different Chloride Solutions Representing Concrete Environments. Mater. Sci. Forum.

[48] Abbas Y., Have B., Hoekstra G.I., Douma A., de Bruijn D., Olthuis W., van den Berg A. (2015). Connecting to concrete: Wireless monitoring of chloride ions in concrete structures. Procedia Eng..

[49] Femenias Y.S., Angst U., Elsener B. (2017). pH-monitoring in mortar with thermally-oxidized iridium electrodes. RILEM Tech. Lett..

[50] Femenias Y.S., Angst U., Elsener B. (2017). Monitoring pH in corrosion engineering by means of thermally produced iridium oxide electrodes. Mater. Corros..

[51] Kolar M., Doliška A., Švegl F., Kalcher K. (2010). Tungsten—Tungsten Trioxide Electrodes for the Long-term Monitoring of Corrosion Processes in Highly Alkaline Media and Concrete-based Materials. Acta Chim. Slov..

[52] Gandía-Romero J.M., Campos I., Valcuende M., García-Breijo E., Marcos M.D., Payá J., Soto J. (2016). Potentiometric thick-film sensors for measuring the pH of concrete. Cem. Concr. Compos..

[53] Bhadra S., Tan D.S.Y., Thomson D.J., Freund M.S., Bridges G.E. (2013). A Wireless Passive Sensor for Temperature Compensated Remote pH Monitoring. IEEE Sens. J..

[54] Du R.-G., Hu R.-G., Huang R.-S., Lin C.-J. (2006). In situ measurement of Cl^−^ concentrations and pH at the reinforcing steel/concrete interface by combination sensors. Anal. Chem..

[55] Yu H., Caseres L. (2012). An embedded multi-parameter corrosion sensor for reinforced concrete structures. Mater. Corros..

[56] Dong S.-G., Lin C.-J., Hu R.-G., Li L.-Q., Du R.-G. (2011). Effective monitoring of corrosion in reinforcing steel in concrete constructions by a multifunctional sensor. Electrochim. Acta.

[57] Norris J.O.W., Grattan K.T., Meggitt B.T. (2000). Optical fiber chemical sensors: Fundamentals and applications. Optical Fiber Sensor Technology.

[58] Krohn D., MacDougall T., Mendez A. (2014). Fiber Optic Sensors: Fundamentals and Applications.

[59] Ding L., Li Z., Ding Q., Shen X., Yuan Y., Huang J. (2018). Microstructured optical fiber based chloride ion sensing method for concrete health monitoring. Sens. Actuators B Chem..

[60] Laferrière F., Inaudi D., Kronenberg P., Smith I.F.C. (2008). A new system for early chloride detection in concrete. Smart Mater. Struct..

[61] Nguyen T.H., Venugopala T., Chen S., Sun T., Grattan K.T.V., Taylor S.E., Basheer P.A.M., Long A.E. (2014). Fluorescence based fibre optic pH sensor for the pH 10–13 range suitable for corrosion monitoring in concrete structures. Sens. Actuators B Chem..

[62] McPolin D.O., Basheer P.A.M., Long A.E., Xie W., Sun T., Grattan K.T.V. (2009). Development and Longer Term In Situ Evaluation of Fiber-Optic Sensors for Monitoring of Structural Concrete. IEEE Sens. J..

[63] Khan F.U., Ahmad I. (2016). Review of energy harvesters utilizing bridge vibrations. Shock Vib..

[64] Moosavi S.R., Gia T.N., Nigussie E., Rahmani A.M., Virtanen S., Tenhunen H., Isoaho J. (2016). End-to-end security scheme for mobility enabled healthcare Internet of Things. Future Gener. Comput. Syst..

[65] Nigussie E., Xu T., Potkonjak M. (2015). Securing wireless body sensor networks using bijective function-based hardware primitive. Proceedings of the 2015 IEEE Tenth International Conference on Intelligent Sensors, Sensor Networks and Information Processing (ISSNIP).

[66] Moosavi S.R., Nigussie E., Levorato M., Virtanen S., Isoaho J. (2018). Performance analysis of end-to-end security schemes in healthcare IoT. Procedia Comput. Sci..

[67] Niu Z., Chen J., Xu L., Yin L., Zhang F. (2013). Application of the Environmental Internet of Things on monitoring PM2.5 at a coastal site in the urbanizing region of southeast China. Int. J. Sustain. Dev. World Ecol..

[68] Wang H., Zhang T., Quan Y., Dong R. (2013). Research on the framework of the Environmental Internet of Things. Int. J. Sustain. Dev. World Ecol..

[69] Su X., Shao G., Vause J., Tang L. (2013). An integrated system for urban environmental monitoring and management based on the Environmental Internet of Things. Int. J. Sustain. Dev. World Ecol..

[70] Zanella A., Bui N., Castellani A., Vangelista L., Zorzi M. (2014). Internet of things for smart cities. IEEE Internet Things J..

[71] Kim T., Ramos C., Mohammed S. (2017). Smart City and IoT. Future Gener. Comput. Syst..

[72] Saeed F., Paul A., Rehman A., Hong W.H., Seo H. (2018). IoT-based intelligent modeling of smart home environment for fire prevention and safety. J. Sens. Actuator Netw..

[73] Wang J., Fu Y., Yang X. (2017). An integrated system for building structural health monitoring and early warning based on an Internet of things approach. Int. J. Distrib. Sens. Netw..

[74] Abdelgawad A., Yelamarthi K. (2017). Internet of things (IoT) platform for structure health monitoring. Wirel. Commun. Mob. Comput..

[75] Barsocchi P., Cassara P., Mavilia F., Pellegrini D. (2018). Sensing a city’s state of health: Structural monitoring system by internet-of-things wireless sensing devices. IEEE Consum. Electron. Mag..

[76] Kuzminykh I. (2017). Testing of communication range in ZigBee technology. Proceedings of the 14th International Conference on The Experience of Designing and Application of CAD Systems in Microelectronics (CADSM).

[77] Centenaro M., Vangelista L., Zanella A., Zorzi M. (2016). Long-range communications in unlicensed bands: The rising stars in the IoT and smart city scenarios. IEEE Wirel. Commun..

[78] Su D., Xia Y., Yuan R. (2018). Self-Powered Wireless Sensor Network for Automated Corrosion Prediction of Steel Reinforcement. J. Sens..

[79] Qiao G., Sun G., Hong Y., Liu T., Guan X. (2014). Corrosion in reinforced concrete panels: Wireless monitoring and wavelet-based analysis. Sensors.

[80] Sun G., Yang G., Guo B. (2017). CoCoMo: Toward controllable and reliable corrosion monitoring with a wireless sensor network. Int. J. Distrib. Sens. Netw..

[81] Xu S., Yu J., Niu D., Dong Z., Gao P. (2012). Research on wireless remote monitoring system of the durability for large concrete structures. Adv. Mater. Res..

[82] Erokhina O.V., Brega A. V Intelligent transport technologies in “smart” cities. Proceedings of the 2020 Systems of Signals Generating and Processing in the Field of on Board Communications.

[83] Mishra L., Vikash, Varma S. Internet of things for military applications. Proceedings of the 7th International Conference on Computing for Sustainable Global Development (INDIACom).

[84] Farooq M.S., Riaz S., Abid A., Umer T., Zikria Y.B. (2020). Role of IoT technology in agriculture: A systematic literature review. Electronics.

[85] Nigussie E., Olwal T., Musumb G., Tegegne T., Lemma A., Mekuria F. (2020). IoT-based irrigation management for smallholder farmers in rural sub-Saharan Africa. Procedia Comput. Sci..

[86] Bekkerman R., Bilenko M., Langford J., Bekkerman R., Bilenko M., Langford J. (2012). Scaling up machine learning: Introduction. Scaling up Machine Learning: Parallel and Distributed Approaches.

[87] Cherkassky V., Mulier F. (2007). Learning from Data: Concepts, Theory, and Methods.

[88] Han J., Kamber M., Pei J. (2012). Data Mining: Concepts and Techniques.

[89] Witten I.H., Frank E., Hall M.A. (2011). Data Mining: Practical Machine Learning Tools and Techniques.

[90] Alpaydin E. (2010). Introduction to Machine Learning.

[91] Reich Y. (1997). Machine learning techniques for civil engineering problems. Microcomput. Civ. Eng..

[92] Karbhari V.M., Lee L.S.-W., Karbhari V.M., Ansari F. (2009). Vibration-based damage detection techniques for structural health monitoring of civil infrastructure systems. Structural Health Monitoring of Civil Infrastructure Systems.

[93] Mitchell T. (1997). Machine Learning.

[94] Kanevski M., Timonin V., Pozdnukhov A. (2009). Machine Learning for Spatial Environmental Data: Theory, Applications, and Software.

[95] Marsland S. (2009). Machine Learning: An Algorithmic Perspective.

[96] Murphy K.P. (2012). Machine Learning: A Probabilistic Perspective.

[97] Harris T. (2015). Credit scoring using the clustered support vector machine. Expert Syst. Appl..

[98] Takeda A., Kanamori T. (2014). Using financial risk measures for analyzing generalization performance of machine learning models. Neural Netw..

[99] Kim M.J., Kang D.K. (2010). Ensemble with neural networks for bankruptcy prediction. Expert Syst. Appl..

[100] Di K., Li W., Yue Z., Sun Y., Liu Y. (2014). A machine learning approach to crater detection from topographic data. Adv. Sp. Res..

[101] Dede G., Sazlı M.H. (2010). Speech recognition with artificial neural networks. Digit. Signal Process..

[102] Hsieh W.W. (2009). Machine Learning Methods in the Environmental Sciences: Neural Networks and Kernels.

[103] Taffese W.Z., Devedžic V. (2007). Case-based reasoning and neural networks for real estate valuation. Proceedings of the 25th IASTED International Multi-Conference: Artificial Intelligence and Applications.

[104] Park B., Bae J.K. (2015). Using machine learning algorithms for housing price prediction: The case of Fairfax County, Virginia housing data. Expert Syst. Appl..

[105] Taffese W.Z., Hamid M.Y., Chekima A., Sainarayanan G., Prabhakaran N., Anthony P., Wong F., Dargham J.A., Wei J.T.T., Kin K.T.T. (2006). A survey on application of artificial intelligence in real estate industry. Proceedings of the Third International Conference on Artificial Intelligence in Engineering & Technology.

[106] Lavecchia A. (2015). Machine-learning approaches in drug discovery: Methods and applications. Drug Discov. Today.

[107] Wang G., Lam K.-M., Deng Z., Choi K.-S. (2015). Prediction of mortality after radical cystectomy for bladder cancer by machine learning techniques. Comput. Biol. Med..

[108] Che D., Liu Q., Rasheed K., Tao X., Arabnia H.R., Tran Q.-N. (2011). Decision tree and ensemble learning algorithms with their applications in bioinformatics. Software Tools and Algorithms for Biological Systems.

[109] Vaughan A., Bohac S.V. (2015). Real-time, adaptive machine learning for non-stationary, near chaotic gasoline engine combustion time series. Neural Netw..

[110] Jurado S., Nebot À., Mugica F., Avellan N. (2015). Hybrid methodologies for electricity load forecasting: Entropy-based feature selection with machine learning and soft computing techniques. Energy.

[111] Kialashaki A., Reisel J.R. (2014). Development and validation of artificial neural network models of the energy demand in the industrial sector of the United States. Energy.

[112] Alom M.Z., Taha T.M., Yakopcic C., Westberg S., Sidike P., Nasrin M.S., Hasan M., Van Essen B.C., Awwal A.A.S., Asari V.K. (2019). A State-of-the-Art Survey on Deep Learning Theory and Architectures. Electronics.

[113] Taffese W.Z., Nigussie E., Isoaho J. (2019). Internet of things based durability monitoring and assessment of reinforced concrete structures. Procedia Comput. Sci..

[114] Taffese W.Z., Al-Neshawy F., Sistonen E., Ferreira M. Optimized neural network based carbonation prediction model. Proceedings of the International Symposium Non-Destructive Testing in Civil Engineering (NDTCE 2015).

[115] Taffese W.Z., Sistonen E., Puttonen J. (2015). Prediction of concrete carbonation depth using decision trees. Proceedings of the 23rd European Symposium on Artificial Neural Networks, Computer Intelligence Machine Learning.

[116] Taffese W.Z., Sistonen E., Puttonen J. (2015). CaPrM: Carbonation prediction model for reinforced concrete using machine learning methods. Constr. Build. Mater..

[117] Kellouche Y., Boukhatem B., Ghrici M., Tagnit-Hamou A. (2019). Exploring the major factors affecting fly-ash concrete carbonation using artificial neural network. Neural Comput. Appl..

[118] Lu C., Liu R. (2009). Predicting carbonation depth of prestressed concrete under different stress states using artificial neural network. Adv. Artif. Neural Syst..

[119] Cho H.-C., Ju H., Oh J.-Y., Lee K.J., Hahm K.W., Kim K.S. (2016). Estimation of concrete carbonation depth considering multiple influencing factors on the deterioration of durability for reinforced concrete structures. Adv. Mater. Sci. Eng..

[120] Luo D., Niu D., Dong Z., Olek J., Weiss J. (2014). Application of neural network for concrete carbonation depth prediction. Proceedings of the 4th International Conference on the Durability of Concrete Structures.

[121] Ghafoori N., Najimi M., Sobhani J., Aqel M.A. (2013). Predicting rapid chloride permeability of self-consolidating concrete: A comparative study on statistical and neural network models. Constr. Build. Mater..

[122] Gilan S.S., Jovein H.B., Ramezanianpour A.A. (2012). Hybrid support vector regression—Particle swarm optimization for prediction of compressive strength and RCPT of concretes containing metakaolin. Constr. Build. Mater..

[123] Peng J., Li Z., Ma B. (2002). Neural network analysis of chloride diffusion in concrete. J. Mater. Civ. Eng..

[124] Inthata S., Kowtanapanich W., Cheerarot R. (2013). Prediction of chloride permeability of concretes containing ground pozzolans by artificial neural networks. Mater. Struct..

[125] Boğa A.R., Öztürk M., Topçu İ.B. (2013). Using ANN and ANFIS to predict the mechanical and chloride permeability properties of concrete containing GGBFS and CNI. Compos. Part B Eng..

[126] Yasarer H., Najjar Y.M. (2014). Characterizing the permeability of Kansas concrete mixes used in PCC pavements. Int. J. Geomech..

[127] Taffese W.Z., Al-Neshawy F., Piironen J., Sistonen E., Puttonen J. (2014). Monitoring, evaluation and long-term forecasting of hygrothermal performance of thick-walled concrete structure. Proceedings of the OECD/NEA WGIAGE Workshop on the Non-Destructive Evaluation of Thick-Walled Concrete Structures.

[128] Taffese W.Z., Sistonen E. (2016). Neural network based hygrothermal prediction for deterioration risk analysis of surface-protected concrete façade element. Constr. Build. Mater..

[129] Song H.-W., Kwon S.-J. (2009). Evaluation of chloride penetration in high performance concrete using neural network algorithm and micro pore structure. Cem. Concr. Res..

[130] Kim Y.-Y., Lee B.-J., Kwon S.-J. (2014). Evaluation technique of chloride penetration using apparent diffusion coefficient and neural network algorithm. Adv. Mater. Sci. Eng..

[131] Hodhod O.A., Ahmed H.I. (2013). Developing an artificial neural network model to evaluate chloride diffusivity in high performance concrete. HBRC.

[132] Parichatprecha R., Nimityongskul P. (2009). Analysis of durability of high performance concrete using artificial neural networks. Constr. Build. Mater..

[133] Taffese W.Z., Sistonen E. (2017). Significance of chloride penetration controlling parameters in concrete: Ensemble methods. Constr. Build. Mater..

[134] Taffese W.Z., Sistonen E. (2017). Machine learning for durability and service-life assessment of reinforced concrete structures: Recent advances and future directions. Autom. Constr..

[135] Hunkeler F., Böhni H. (2005). Corrosion in reinforced concrete: Processes and mechanisms. Corrosion in Reinforced Concrete Structures.

[136] Akid R., Wessel J.K. (2004). Corrosion of engineering materials. The Handbook of Advanced Materials: Enabling New Designs.

[137] Barroca N., Borges L.M., Velez F.J., Monteiro F., Górski M., Castro-Gomes J. (2013). Wireless sensor networks for temperature and humidity monitoring within concrete structures. Constr. Build. Mater..

[138] Cabezas J., Sánchez-Rodríguez T., Gómez-Galán J.A., Cifuentes H., Carvajal R.G. (2018). Compact embedded wireless sensor-based monitoring of concrete curing. Sensors.

[139] Strangfeld C., Johann S., Bartholmai M. (2019). Smart RFID sensors embedded in building structures for early damage detection and long-term monitoring. Sensors.

